# The Influence of Various Photoinitiators on the Properties of Commercial Dental Composites

**DOI:** 10.3390/polym13223972

**Published:** 2021-11-17

**Authors:** Andrea Kowalska, Jerzy Sokolowski, Tomasz Gozdek, Michał Krasowski, Karolina Kopacz, Kinga Bociong

**Affiliations:** 1 Department of General Dentistry, Medical University of Lodz, 92-213 Lodz, Poland; jerzy.sokolowski@umed.lodz.pl (J.S.); kinga.bociong@umed.lodz.pl (K.B.); 2 Institute of Polymer & Dye Technology, Lodz University of Technology, Stefanowskiego 12/16, 90-924 Lodz, Poland; tomasz.gozdek@p.lodz.pl; 3 University Laboratory of Materials Research, Medical University of Lodz, Pomorska 251, 92-213 Lodz, Poland; michal.krasowski@umed.lodz.pl; 4 “DynamoLab” Academic Laboratory of Movement and Human Physical Performance, Medical University of Lodz, Pomorska 251, 92-215 Lodz, Poland; karolina.kopacz@umed.lodz.pl; 5 Department of Health Sciences, Medical University of Mazovia, Rydygiera 8, 01-793 Warszawa, Poland

**Keywords:** camphorquinone, Lucirin TPO, Ivocerin, RAP technology^®^, resin-based composite, dental composites, commercial composite, restorative dentistry, preventive dentistry

## Abstract

The aim of this article was to compare the biomechanical properties of commercial composites containing different photoinitiators: Filtek Ultimate (3M ESPE) containing camphorquinone (CQ); Estelite Σ Quick (Tokuyama Dental) with CQ in RAP Technology^®^; Tetric EvoCeram Bleach BLXL (Ivoclar Vivadent AG) with CQ and Lucirin TPO; and Tetric Evoceram Powerfill IVB (Ivoclar Vivadent AG) with CQ and Ivocerin TPO. All samples were cured with a polywave Valo Lamp (Ultradent Products Inc.) with 1450 mW/cm^2^. The microhardness, hardness by Vicker’s method, diametral tensile strength, flexural strength and contraction stress with photoelastic analysis were tested. The highest hardness and microhardness were observed for Filtek Ultimate (93.82 ± 17.44 HV), but other composites also displayed sufficient values (from 52 ± 3.92 to 58,82 ± 7.33 HV). Filtek Ultimate not only demonstrated the highest DTS (48.03 ± 5.97 MPa) and FS (87.32 ± 19.03 MPa) but also the highest contraction stress (13.7 ± 0.4 MPa) during polymerization. The TetricEvoCeram Powerfill has optimal microhardness (54.27 ± 4.1 HV), DTS (32.5 ± 5.29 MPa) and FS (79.3 ± 14.37 MPa) and the lowest contraction stress (7.4 ± 1 MPa) during photopolymerization. To summarize, Filtek Ultimate demonstrated the highest microhardness, FS and DTS values; however, composites with additional photoinitiators such as Lucirin TPO and Ivocerin have the lowest polymerization shrinkage. These composites also have higher FS and DTS and microhardness than material containing CQ in Rap Technology.

## 1. Introduction

Modern medicine not only includes life-saving treatments but also aesthetic procedures. This trend can be also observed in modern dentistry. Patients pay attention to the appearance of a tooth filling, and the dentist must face choosing a suitable color and creating a tooth-like shape. The dentist must also select a suitable dental composite with optimal biomechanical features. However, the shade of composite depends on the amount and size of filler and the type of photoinitiators [1] and can change in the oral cavity environment due to the effect of saliva, beverages, food and nicotine [2].

The most common photoinitiator system in dental composite is camphorquinone (CQ) and its co-initiator tertiary amines [3]. CQ is an alpha-diketone in the form of a yellow powder; its absorbance ranges from 360 to 510 nm, and its maximum absorbance is 468 nm [4,5,6]. The tertiary amines are used to increase efficiency in the production of free radicals [7]. However, CQ has many disadvantages as a photoinitiator: poor bleaching properties, yellowish staining and is toxic [4,5]. To reduce the amount of the CQ/amines, RAP Technology^®^ (radical amplified photopolymerization) has been developed, in which the CQ is recycled during the generation of the initiator: A single molecule can, hence, produce multiple radicals. In this technology, CQ is more reactive than in standard layout CQ/amines [8].

CQ and its co-initiators are not the only photoinitiator system used in commercial dental composites. Lucirin TPO, used by Ivoclar Vivadent, or diphenyl(2,4,6-trimethylben-zoyl)phosphin oxide, does not require co-initiators to start polymerization process [9]. The range of absorption of Lucirin TPO is 380–425 nm, and the maximum absorbance is 400 nm [10]. This photoinitiator is a colorless liquid and produces less yellowish polymers than CQ [11]. It is also more effective than CQ due to its ability to produce two free radicals by alpha-cleavage, which starts the polymerization process immediately [9]. Alternatively, Ivoclar Vivadent has also developed Ivocerin: a dibenzoyl germanium derivative. Its absorption range is 370 to 460 nm [12], and its absorbance maximum is 418 nm [13]. Similarly to other germanium derivatives, Ivocerin forms at least two radicals. It can be relatively easy synthesized, has low cytotoxicity and it is not mutagenic [13].

The main photoinitiator system used in commercial composites comprises CQ with tertiary amines. There is evidence that changing the action of CQ to RAP Technology^®^ may increase the degree of curing [14]. Moreover, the samples demonstrate generally the same level of hardness throughout when irradiated for at least 10 s [8]. Other photoinitiators are also used, but they have a significant influence on the properties of dental resins. According to Salgado et al., using only TPO reduces the depth of curing but increases surface hardness; in addition, using CQ with TPO increases the depth of curing and top hardness [15]. It has also been found that using TPO instead of CQ does not influence the degree of conversion even though TPO produces more free radicals. This lack of effect can be caused by using a mismatched dental curing lamp with a narrow light range [16]. Ivocerin also significantly affects the mechanical features of the filling. Alkhudhairy et al. [12] noted that luting cements containing Ivocerin produce deeper cures. Delgado et al. [9] reports that dental composites including Ivocerin have higher values of flexural strength and ultimate tensile strength than composites containing CQ and that resins including a combination of TPO and Lucirin as a photoinitiator system demonstrate the highest flexural strength and ultimate tensile strength.

The aim of this study is to compare properties of commercial composites containing different photoinitiators. The null hypothesis is that the use of additional photoinitiators with CQ, such as Lucirin TPO, Ivocerin or radical amplified photopolymerization of CQ (RAP Technology), influences microhardness, flexural strength, diameter tensile strength and contraction stress of the composite.

## 2. Materials and Methods

The composites are shown in Table 1. Filtek Ultimate is used as a control group due to its popularity as a research object and very good mechanical properties.

The materials were cured with a polywave Valo Lamp (Ultradent Products Inc., South Jordan, UT, USA) with three irradiance outputs (1000 mW/cm^2^, 1450 mW/cm^2^ and 3200 mW/cm^2^) and a light range of 395–510 nm. The lamp was calibrated with a Digital Light Meter 200 radiometer system (Rolence Enterprice Inc., Taoyuan City, Taiwan) to ensure consistent irradiance. The material was irradiated according to lamp manufacturer’s instruction for 8 s per each side (top and bottom of sample) in silicon molds placed between two microscope slides. The samples used for hardness and diametral tensile strength testing were cylindrical (3 mm tall and 6 mm diameter). The silicon molds were filled with dental composite, and the material was irradiated on one or both sides depending on type of examination.

**Figure 1:**
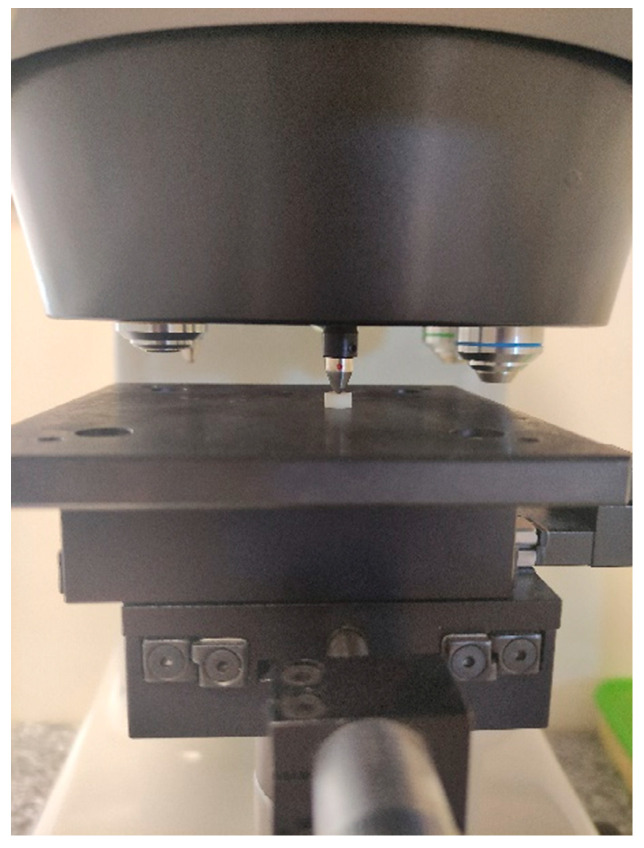
The sample during Vicker’s hardness test.

The Vickers hardness (HV) of the dental composite was determined with a semi-automatic hardness tester (ZHV2-m Zwick/Röell, Ulm, Germany) (Figure 1). A square-based diamond pyramid with an apex angle of 136° is used; the indenter was loaded with 1000 g, and the contact time was 10 s. In order to define mean HV, the samples were irradiated on both sides. In order to measure the hardness at the top, bottom and 1.5 mm below the surface, the samples were exposed to the light emitter only on one side (top of the sample).

**Figure 2:**
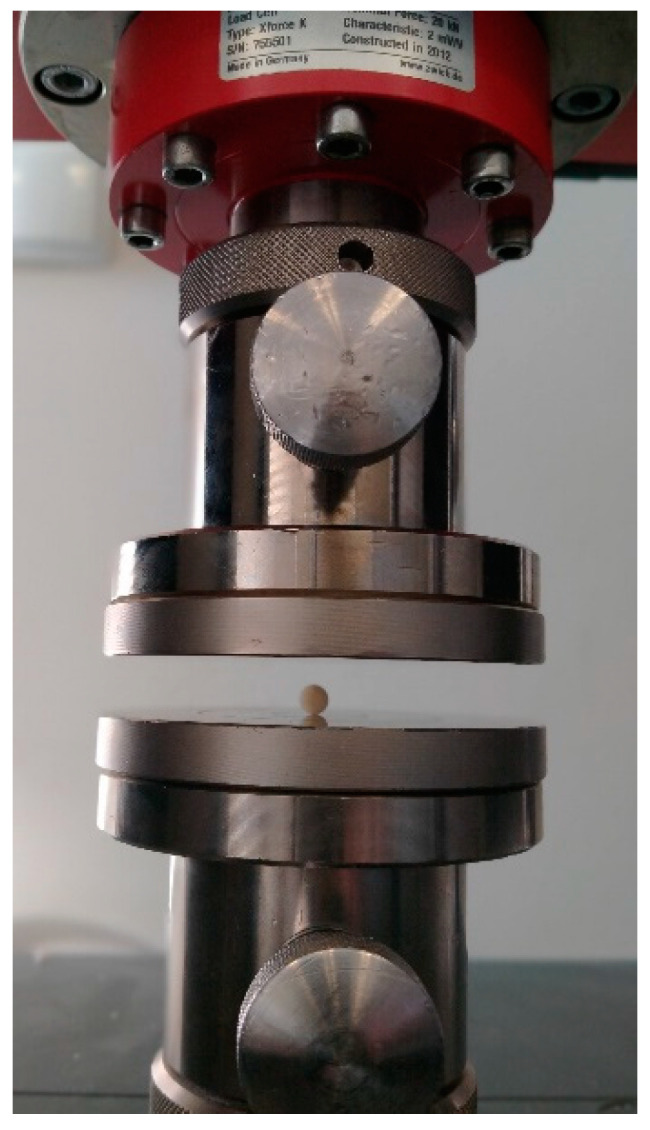
The sample during diametral tensile test.

The diametral tensile strength test (DTS) assesses the strength of the dental composite materials. DTS is the maximum resistance against loads tending to destroy a sample. The crosshead speed was 2 mm/min (Figure 2). The test was performed on universal testing machine (Z020, Zwick/Röell, Ulm, Germany). The numerical value is calculated according to the following formula:

(1) $$\mathrm{DTS}\;[\mathrm{MPa}]=\frac{P\;}{S}\;\left[\frac{N}{{\mathrm{mm}}^{2\;}}\right]=\frac{P}{\frac{1}{2}\;\left(2\pi \frac{D}{2}T\right)}=\frac{2P}{\pi \mathrm{DT}}\left[\frac{N}{{\mathrm{mm}}^{2}}\right]$$

where

DTS—diametral tensile strength (MPa);

P—load applied (N);

S—surface (mm^2^);

D—diameter of sample (mm);

T—high of sample (mm).

**Figure 3:**
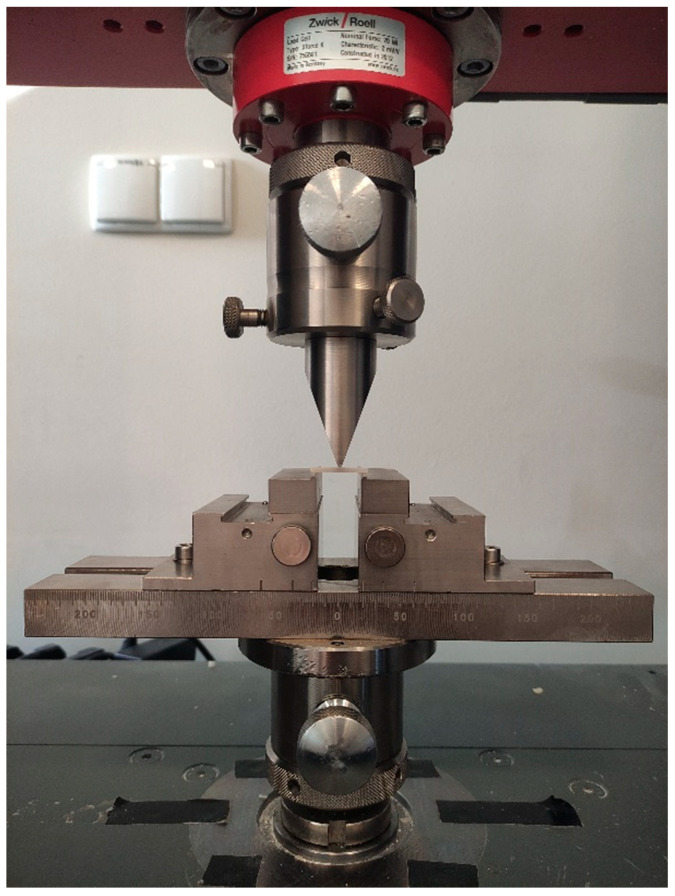
The sample during three-point bending test, evaluated on a universal testing machine.

Flexural strength was evaluated in a three-point bending test. The samples used in this test were rectangular (25 × 2 × 2 mm), and they were irradiated at three points twice for 4 s on both sides: In total, the sample was irradiated for 48 s. The samples were then placed on two supports 20 mm apart. A force was applied in the middle, downwards at a 90° angle (Figure 3). The test was performed on a universal testing machine (Zwick Z020, Zwick/Röell, Ulm, Germany), the crosshead speed was 1 mm/min and the examination complied with ISO regulations [17]. The maximum force, which destroyed the sample, was measured for each specimen.

Flexural strength (MPa) was defined with following equation:

(2) $$\mathrm{FS}\;[\mathrm{MPa}]=\frac{3\mathrm{Wl}}{2{\mathrm{bh}}^{2}}$$

where

W—force, which caused the destruction of the sample (N);

l—distance between supports, 20 mm; 

b—width of sample (mm);

h—high of sample (mm).

**Figure 4:**
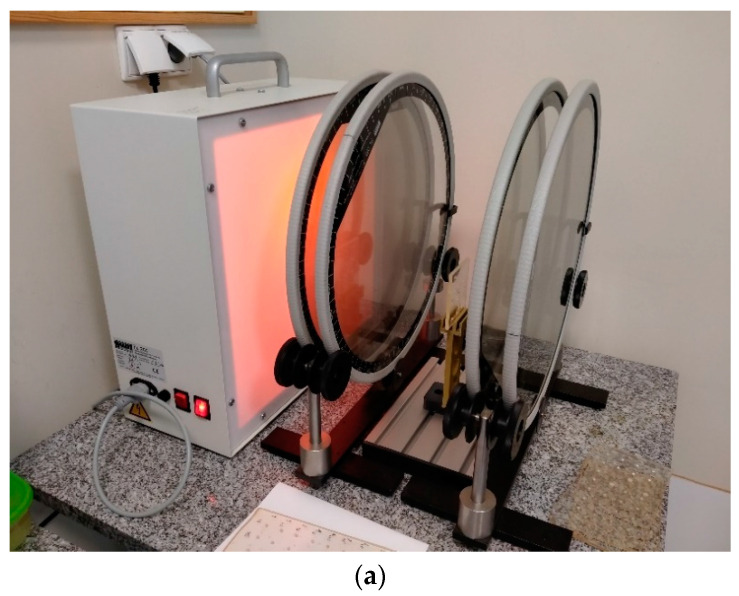

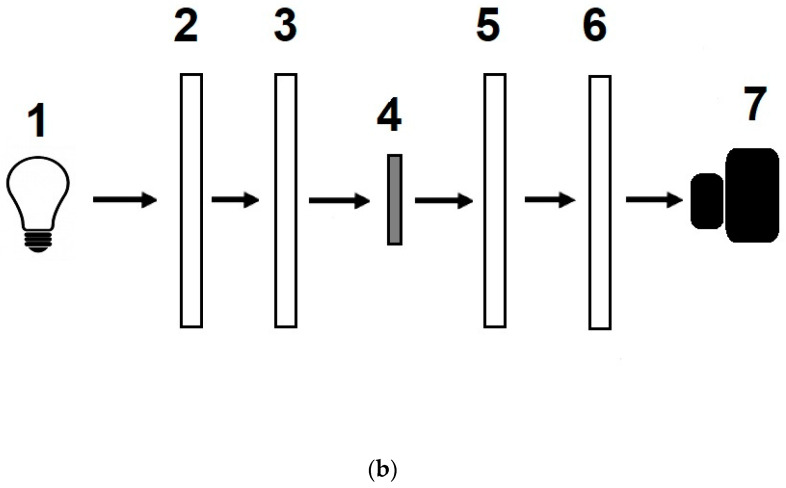
(**a**) Polariscope FL200 Gunt, Germany. (**b**) The photoelastic analysis scheme. 1—light source; 2—polarizer; 3—quarter-wave plate; 4—samples (photoelastically sensitive plates made of epoxy resin filled with composites); 5—quarter-wave plate; 6—analyzer; 7—digital camera.

Photoelastic analysis allows quantitative measurement and visualization of the stress concentration that occurs during photopolymerization (cf. [18,19,20]). Photoelastically sensitive plates of epoxy resin (Epidian 53, Organika-Sarzyna SA, Nowa Sarzyna, Poland) were used in this study. These plates become optically double-refractive under stress. Calibrated orifices 3 mm in diameter and 4 mm thick were prepared in resin plates to mimic a tooth cavity and average size clinical conditions. The orifices were sandblasted with a 50 μm grain corundum Cobra (Renfert, Hilzingen, Germany), and a dedicated bonding system was applied and cured with Valo lamp. This process was conducted to ensure bonding. The holes were filled with selected material in one layer and then cured with the Valo lamp. Three samples were prepared for each commercial material. After 24 h, the generated strains in the plates were visualized using a FL200 circular transmission polariscope (Gunt, Barsbüttel, Germany) (Figure 4). Photoelastic images were recorded by a Canon EOS 5D Mark II digital camera (Canon Inc., Tokyo, Japan) both in parallel and perpendicular to the orientation of the filter polarization planes.

The arrangement and dimensions of the interference fringes were determined using Met-Ilo software. Stress intensity around composite filling was calculated based on the relevant equations. The analysis of stress and strain was carried out in a two-dimensional state of the stresses and three-dimensional state of deformations. Additionally, the calculation was conducted assuming that the relative change in volume of the composite material causes both its extension and that of the base material, i.e., the “tooth model” (epoxy resin plate). Therefore, photoelastic strain calculations were based on the modified Timoshenko’s Equations (3a) and (3b) [21]:

(3a) $$\sigma_{r}=\frac{a^{2}\cdot p_{s}}{b^{2}-a^{2}}\cdot \left(\frac{b^{2}}{r^{2}}-1\right)$$

(3b) $$\sigma_{\theta }=\frac{a^{2}\cdot p_{s}}{b^{2}-a^{2}}\cdot \left(\frac{b^{2}}{r^{2}}+1\right)$$

where

σ_r_—is radial stress (MPa);

σ_θ_—is circumferential stress (MPa);

p*_s_*—is the shrinkage stress around composite filling (MPa);

a—is the radius of the internal orifices in the plate (mm);

b—is the radius of the largest of isochromatic fringes (mm);

r—is the radius contained in the region from a to b.

**Figure 5:**
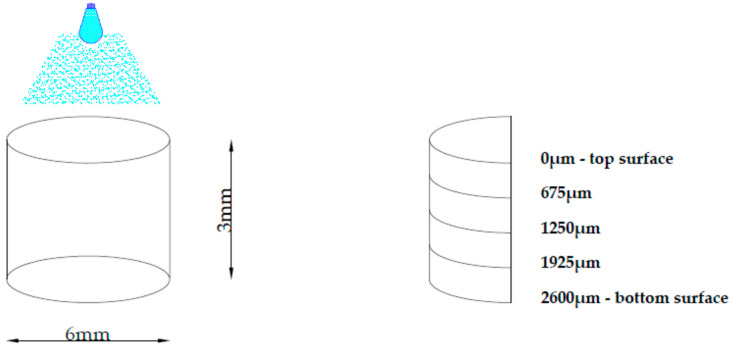
Schematic diagram of the cylindrical sample (diameter of 6 mm and thickness of 3 mm) used for the microhardness test. The top surface was cured twice for 4 s. The microhardness and reduced modulus of the external surfaces (0 µm—top sample’s surface; 2600 µm—bottom sample’s surface) and cross section (675, 1250 and 1925 µm) of the sample were tested.

The microhardness of the composites was tested with the NanoTest 600 (Micromaterials Ltd., Wrexham, Great Britain) using a Berkovich indenter. The maximum force was 10 mN, and the loading and unloading speed was dP/dt = 0.5 mN/s. The measurements (microhardness and reduced modulus) were carried out in controlled conditions of temperature (T = 20 °C) and relative humidity (60 ± 5%). The composite microhardness and reduced modulus was calculated on the basis of the unloading curve, according to Olivier and Pharr [22]. The microhardness and reduced modulus were tested on the external surfaces (0 and 2600 µm) of the sample and on the cross section (675, 1250 and 1925 µm) (Figure 5).

Statistical analysis was performed by using Excel 2010 (Microsoft) and Statistica v. 13 software (Statsoft, Krakow, Poland). The Shapiro–Wilk test of normality was applied for continuous variables. Further analyses were performed by using the Kruskall–Wallis test in the case of a non-normal distribution. In case of normal distribution, the equality of variances was assessed with Levene’s test: in the case of equal variances, ANOVA with Scheffe’s post hoc test was applied. The accepted level of significance was α = 0.05.

## 3. Results

**Figure 6:**
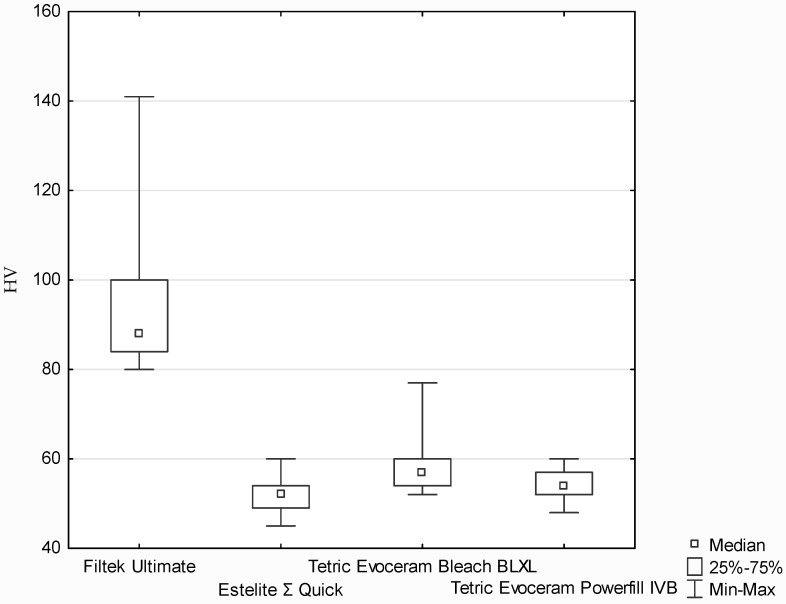
Vicker’s hardness of composites: samples were cured on both sides.

**Figure 7:**
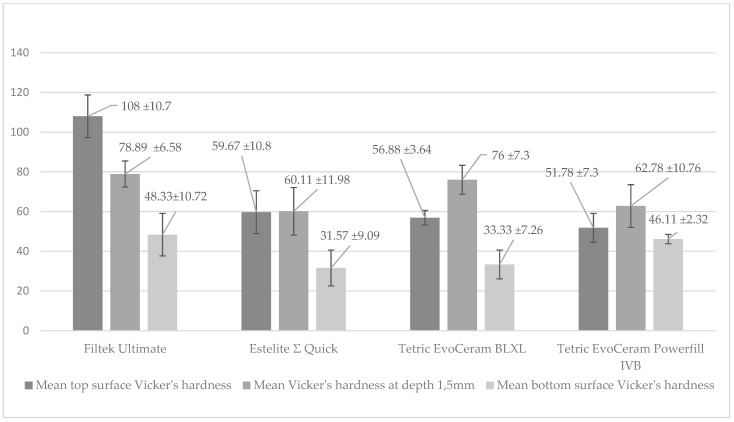
Vicker’s hardness scores of the commercial composites; samples were cured on one side—top of the sample.

The Vicker’s hardness test results are shown in Figure 6 and Figure 7. According to the Kruskal–Wallis test, a statistically significant difference was demonstrated in the hardness on the top surface (*p*-value = 0.0014). Based on the post hoc test of multiple comparisons of mean ranks for all trials, statistically significant differences were found between Filtek Ultimate and the following three types: Estelite Σ Quick (*p*-value = 0.01957), Tetric Evoceram Bleach BLXL (*p*-value = 0.00580) and Tetric Evoceram Powerfill IVB (*p*-value = 0.00002) (Figure 6).

All composites demonstrated similar mean Vicker’s hardness values (samples cured both sides), but the best values were obtained by Filtek Ultimate. At the top surface, the highest results were obtained by Filtek Ultimate (these samples were cured only one side); composites containing different photoinitiator systems have mostly the same hardness at the top. The highest hardness inside the sample (1.5 mm depth) was demonstrated also by Filtek Ultimate and the lowest by Estelite ∑ Quick. Tetric Evoceram Powerfill IVB and Estelite Σ Quick have similar values. At the bottom surface, the highest hardness was observed for Filtek Ultimate. 

Mean Vicker’s hardness was measured on samples that were cured on two sides; other Vicker’s hardness on cross section and microhardness were tested on samples cured on one side.

Microhardness was also tested by nanoindentation (Table 2). At the top surface, the highest microhardness was observed for Tetric EvoCeram Powerfill. At distances of 675 µm and 1250 µm, significantly greater hardness was observed for Filtek Ultimate. However, the two Tetric composites demonstrated quite similar hardness values inside the cross section of sample. In addition, the greatest microhardness values were noted for Tetric EvoCeram Powerfill then for Tetric EvoCeram BLXL at the deeper layer (1925 µm).

The smallest changes in microhardness over distance were observed for Tetric EvoCeram Powerfill. The lowest microhardness values were demonstrated by Estelite Σ Quick. Of note, all resins showed a dramatic reduction in microhardness in the lowest layers. 

**Figure 8:**
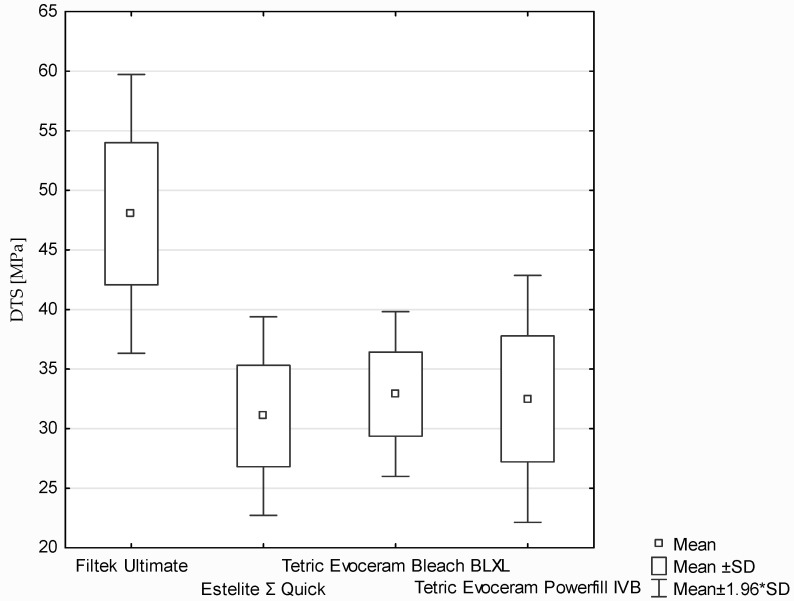
Diametral tensile strength (MPa) between composites.

The strength of dental composite was determined based on diametral tensile strength (Figure 8). According to the ANOVA test, a statistically significant difference was demonstrated in the DTS (*p*-value = 0.00000). Statistically significant differences were found between the following pairs of samples (post hoc Scheffe test; Figure 8):

‑ Filtek Ultimate and Estelite Σ Quick (p-value = 0.00000);
‑ Filtek Ultimate and Tetric Evoceram Bleach BLXL (p-value = 0.00000);
‑ Filtek Ultimate and Tetric Evoceram Powerfill IVB (p-value = 0.00000).

**Figure 9:**
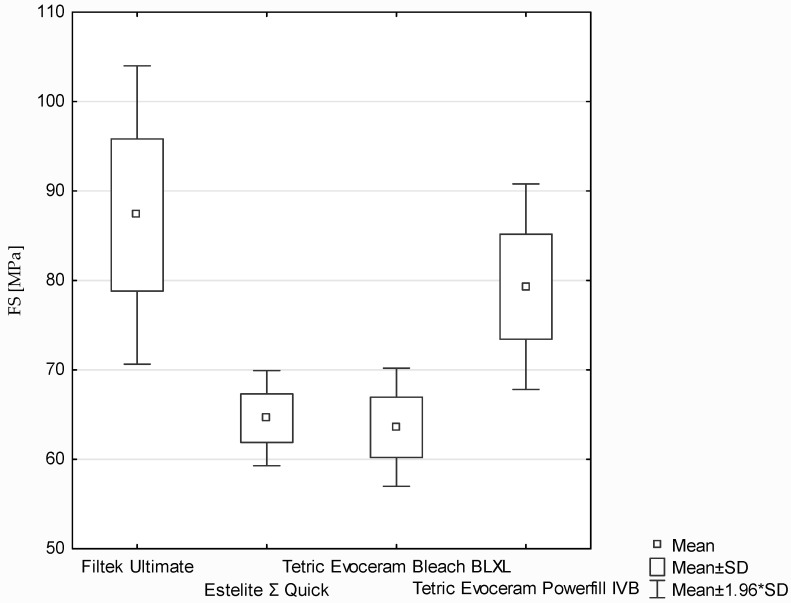
The results of flexural strength (MPa) between composites.

The flexural strength of the dental composites was determined based on the three-point bending test (Figure 9). According to the ANOVA test, a statistically significant difference was demonstrated in flexural strength (*p*-value = 0.01797). Statistically significant differences were found between the following (post hoc Scheffe test: Figure 9):

‑ Filtek Ultimate and Estelite Σ Quick (*p*-value = 0.03979);
‑ Filtek Ultimate and Tetric Evoceram Bleach BLXL (*p*-value = 0.03969).

**Figure 10:**
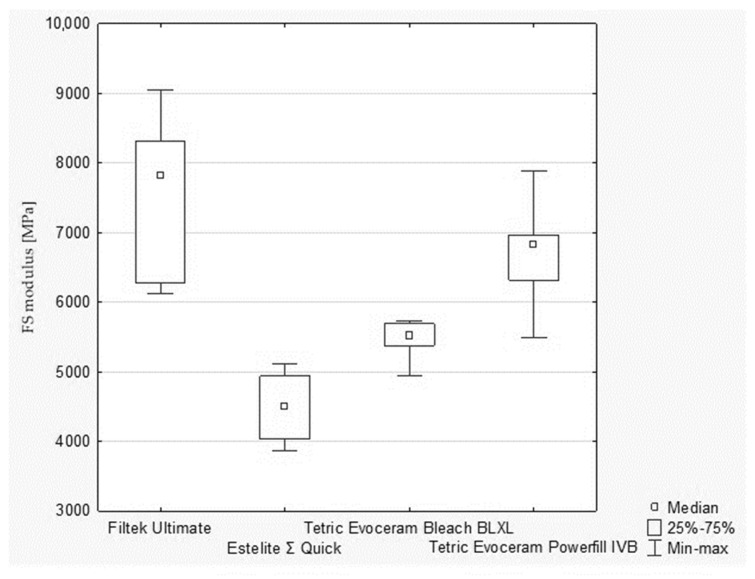
The results of FS modulus (MPa) between composites.

During evaluation of flexural strength also the modulus of elasticity in bending was tested. According to the Kruskal–Wallis test, a statistically significant difference was demonstrated in the FS modulus (*p*-value = 0.0009). Statistically significant differences were found between the following pairs of samples (post hoc test of multiple comparisons of mean ranks for all trials; Figure 10):

‑ Filtek Ultimate and Estelite Σ Quick (*p*-value = 0.002116);
‑ Estelite Σ Quick and Tetric Evoceram Powerfill IVB (*p*-value = 0.00761).

All mechanical properties are summarized in the Appendix A (Table A1).

**Figure 11:**
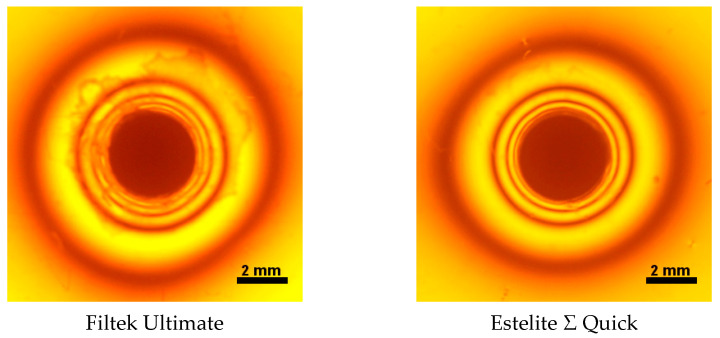

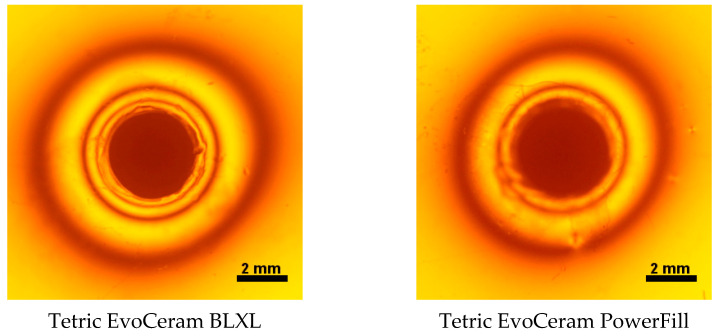
The isochromatic patterns around tested fillings in Epidian 53 resin plate observed under polarized sodium light parallel to polarization plane.

The photoelastic analysis (Table 3) found Filtek Ultimate to have the highest contraction stress during photopolymerization (σint) while Tetric EvoCeram PowerFill had the lowest. Some of the Filtek Ultimate samples tore off from the epoxy resin plates during the photoelastic test; however, this was not observed for the other composites. The results are given in Figure 11. The Kruskal–Wallis test revealed no statistically significant differences between Estelite Σ Quick, Tetric Evoceram Bleach BLXL and Tetric Evoceram Powerfill IVB (*p*-value = 0.1653).

## 4. Discussion

The present article examines the effects of different types of photoinitiator on the mechanical properties of dental composites. It uses a range of analyses that highlight the most important features of dental resins. The null hypothesis of our research confirmed that additional photoinitiators such as Lucirin TPO or Ivocerin TPO affect the properties of composites.

The Vicker’s hardness testing method is a universal and simple method for evaluate the quality of a material and charges in hardness resulting from curing. However, a more specific method is to assess hardness by nanoindentation: this method shows the microhardness of materials according to the depth of sample expressed in micrometers.

Of the tested materials, Filtek Ultimate, which contains camphoriquinone and tertiary amines as a photoinitiator system, demonstrated the highest hardness and microhardness. A significant difference was observed between Filtek Ultimate and Estelite Σ Quick, which could be caused by the filler composition: Filtek Ultimate contains zirconium as a filler, which is a strong material. This study focuses mostly on photoinitiator systems, but it should be underlined that the other components of dental composites have significant influence on the properties of these materials. The zirconium not only improves the hardness of the material but also DTS and FS [23]. Ludovicchetti et al. [24] also report that materials containing zirconium have higher microhardness and high wear resistance, which may result in lower surface roughness. Estelite Σ Quick is called “quick-cure” and although it contains CQ, it uses RAP Technology^®^. It does not have high hardness and, hence, is not abrasive to opposing teeth. 

Ilie et al. [8] noted that the hardness of the composites depends on the time of irradiation. When the samples were irradiated for 20 s, the values of hardness were higher, especially in the deeper layers. During our analysis, all samples were irradiated for 8 s; hence, they demonstrated lower hardness. Although the Estelite Σ Quick manufacturer states that the time of irradiation can be reduced to 60%, longer irradiation periods seem justified.

It is important to note that all samples were irradiated with the same lamp at the same parameters. According to the data provided by the lamp producer, to cure a 2 mm layer of dental composite, it is enough to irradiate the surfaces twice for 4 s with a power of 1450 mW/cm^2^. In this study, the samples were irradiated according to the recommendations of the Valo lamp manufacturer rather than those of the composite producer.

Significant differences of hardness were observed between Estelite Σ Quick and Tetric Evoceram Powerfill IVB. Tetric EvoCeram Powerfill IVB contains three types of photoinitiator, TPO, Ivocerin and gold mean CQ/amines [25], and our findings indicate that it demonstrated sufficient microhardness in every layer apart from the lowest one. Similarly, it has been found that this material needs longer irradiation in order to improve hardness and degree of conversion; a 3 s light curing protocol was insufficient [26]. In the present study, irradiation time had the same influence on Estelite Σ Quick and Tetric Evoceram Powerfill IVB.

Tetric EvoCeram Bleach XL has slightly better microhardness than Tetric Evoceram Powerfill IVB. These composites have similar fillers to each other, and they do not contain zirconium compounds; as such, the fillings can have lower wear resistance than conventional composites [27].

The diametral tensile strength results were similar to the microhardness results. Statistically significant differences were observed between Filtek Ultimate and Estelite Σ Quick. Filtek Ultimate has many advantages: It is wear-resistant, has a good gloss and has significantly higher fracture toughness than other well-known dental composites. Our findings confirm that Filtek Ultimate has significantly higher DTS scores than the other composites. In addition, Filtek Ultimate demonstrated higher flexural strength, as also noted by the manufacturers’ own analysis [28].

Estelite Σ Quick includes CQ based on RAP Technology. RAP Technologies’ new catalyst technology was discovered in 2005, which can remarkably accelerate the curing process. During curing, CQ is excited by irradiation and releases hydrogen from the alpha-position, resulting in the formation of amine-derived radicals. In this technology CQ is consumed and CQ can only produce one initiator molecule. In RAP Technology^®^, the irradiation of CQ is caused by a light, and the excited CQ transfers the energy to radical amplifier. Next, the radical amplifier is excited and reacts with monomers to produce polymers. After transferring the energy to the radical amplifier, CQ returns to ground state, and it can be once again irradiated. The CQ in this technology is recycled, and it can produce multiple radicals [29].

Another statistically significant difference was found between Filtek Ultimate and Tetric EvoCeram Bleach XL. The Tetric EvoCeram Bleach XL composite is a nanohybrid and contains limited amounts of CQ, which has been replaced by Lucirin TPO [30]. Lucirin TPO is more efficient than CQ due to its large photo absorption efficiency. TPO also produces more free radicals because it undergoes alpha-cleavage [31]; briefly, the compound breaks down and undergoes rapid cleavage and yields two radicals [32,33]. Using TPO also improves the mechanical properties and degree of conversion of material [32].

In our analysis, the microhardness results for Tetric EvoCeram Bleach XL and the DTS and FS results for Tetric EvoCeram Bleach XL are sufficient but lower than those of Filtek Ultimate. No expected improvement was observed despite the presence of TPO, probably due to the duration of light irradiation. Maybe curing the composites for 10 s or more will improve the values of above-mentioned tests, but this requires further analysis. Although the dental lamp used in this study has a wide range of light (390–510 nm), the absorbance range of Lucirin TPO is lower at 380–420 nm. Some sources indicate that the maximum absorbance of TPO is at 380 nm [34]; however, these values are out of the range of the lamp. Hence, this could have resulted in slower polymerization and could have influenced the hardness, DTS and FS readings. Tetric EvoCeram Powerfill IVB also has better DTS values than Tetric EvoCeram Bleach XL. Both Tetric materials also yielded satisfying photoelastic results; this could be due to the fact that both CQ and Lucirin TPO were used as photoinitiators and that the polymerization was delayed.

Estelite Σ Quick has slightly lower DTS than Tetric EvoCeram Bleach. Similarly, in a previous study, the mean DTS of Estelite Σ Quick was found to be 35.95 (±1.070) MPa with a 650 mW/cm^2^ lamp; 44.81 (±1.2) MPa with a 1200 mW/cm^2^ lamp; 38.15 (±2.1) MPa for Tetric EvoCeram Bulkfill with a 650 mW/cm^2^ lamp; and 48.8 (±1.8) MPa with a 1200 mW/cm^2^ lamp [35]. These results are higher than those in the present study, probably due to the shorter irradiation time (8 s per side). These findings confirm that longer exposure for UV light yields higher DTS.

Filtek Ultimate has significantly higher values of flexural strength (FS) than Estelite Σ Quick. Filtek Ultimate is known to have very high values of FS. Thomaidis et al. reported a FS value of 103 MPa when the sample was cured 40 s for both sides with lamp power 950 mW/cm^2^ [36]. Additionally, Tetric EvoCeram Powerfill has also higher values of FS than Estelite Σ Quick. In a previous study, Estelite Σ Quick samples cured at 1100 mW/cm^2^ for 20 s in three points were found to yield FS values of 97.4 (±10.91), while Tetric EvoCeram bulkfill demonstrated a mean FS value of 116.09 (±14.8) MPa [37].

The FS values of Tetric EvoCeram PowerFill have been found to depend on a light source. A mean FS of 129 MPa was achieved at 2400 mW/cm^2^ for 3 s and 140.9 MPa at 1600 mW/cm^2^ for 20 s [26]. Our present results indicate lower FS values; hence, we recommend extending the irradiation time to be compatible with the composite manufacturers’ instructions.

Isochromatic patterns of the tested composites are given in Figure 11. Although Filtek Ultimate obtained good Vicker’s hardness, DTS and FS results, it also demonstrated the most amount of isochromic patterns. Materials with high contraction stress during polymerization may be particularly prone to deterioration of the bond strength with dentine, resulting in microleakage and secondary caries [38]. Shrinkage stress can rapidly build up during polymerization by replacing the van der Waals spaces by covalent bonds, thus reducing the free volume [35,36]. Shrinkage values are dependent on material composition, cavity configuration and compliance and the viscoelastic nature of material [38,39]. Filtek Ultimate contains smaller amounts of filler compared to other composites and, moreover, contains TEGDMA, which increases the shrinkage values [30]. Similar polymerization shrinkage values were confirmed by Domarecka et al.: a shrinkage stress value of 14.1 (±0.8) MPa was obtained under 1200 mW/cm^2^ for 20 s [40].

Estelite Σ Quick also demonstrated a large number of isochromatic patterns. The material undergoes rapid polymerization; it is sensitive to natural light and some of the samples polymerized under the light of the desk lamp. Rapid polymerization of material can cause high polymerization stress. Our analysis found that conventional composites have higher polymerization shrinkage than bulkfill materials. In contrast, Tetric EvoCeram Powerfill yielded optimal stress results during polymerization. This may be caused by the presence of inhibitors of polymerization. Using the three different types of photoinitiators probably slows the process of polymerization and reduces the generated stress. Thus, this composite provides tight filling and could lengthen the service life of the dental composite in the oral cavity.

As the composites used in this study have different photoinitiator systems, they have various strengths, such as dental fillings. Filtek Ultimate has high hardness, making it suitable for use in posterior teeth especially in first and second Black’s class, but this can be problematic for patients suffering from bruxism. Moreover, its color is not suitable for bleached teeth, and color stability might be doubtful due to it using CQ and tertiary amines, which yellow in color. Filtek Ultimate is not a bulkfill dental composite; thus, it needs to be applied in layers into the cavity; this extends working time. Due its Rap Technology, Estelite Σ Quick is characterized by short working time and the color is more stable due to lower amounts of CQ; it has lower microhardness than Filtek Ultimate and, hence, can be used in patients with temporo-mandibular joint problems. Tetric EvoCeram Bleach XL is the whitest of the tested composites and matches with extremely bleached teeth; it demonstrates good microhardness in most layers and, hence, can used in Black’s class II and I cavities in both front and posterior teeth. Tetric EvoCeram Powerfill IVB is a bulkfill; thus, it can be used in Black’s class I and II, but it can be useless in front teeth due to its translucency; it also has a shorter application times.

## 5. Conclusions

The Filtek Ultimate composite with CQ and tertiary amines as the initiator system serves as a “golden mean”: it has the highest values of microhardness, FS and DTS. However, composites including additional photoinitiators (Tetric EvoCeram Bleach and Tetric Evoceram Powerfill) gained better values than that containing RAP Technology. The highest polymerization shrinkage was observed for Filtek Ultimate. The lowest polymerization stress was observed for composites containing additional photoinitiators, which are also bulkfill.

The composite should be cured according to the manufacturer’s instructions, especially the curing time rather than the values given by the manufacturer of the dental curing unit. It is necessary to use a suitable dental lamp with a light wave range encompassing the range of absorbance of the photoinitiator in order to gain optimal mechanical properties.

## Figures and Tables

**Table 1:** Restorative materials used in the studies.

Restorative Materials	Material Type	Manufacturer	Filler	Polymer Matrix	Photoinitiator
Filtek Ultimate A2	Nanofill	3M ESPE St. Paul, MN, USA	Silica particles, Zirconium particles (78.5 wt%, 63.3 vol%)	Bis-GMA, Bis-EMA, UDMA, TEGDMA, PEGDMA	Camphorquinone, tertiary amines
Estelite Σ Quick A02	Nanofill	Tokuyama Dental, Tokyo, Japan	Composite filler, Silica-zirconia filler (71 vol%)	Bis-GMA, UDMA, TEGDMA	Camphorquinone (RAP Technology^®^)
Tetric EvoCeram Bleach BLXL	Nanohybrid	Ivoclar Vivadent AG, Schaan, Liechtenstein	Barium glass, ytterbium trifluoride, mixed oxide and copolymers (79 wt%, 60 vol%)	Bis-GMA UDMA TEGDMA	Camphorquinone, Lucirin TPO
Tetric Evoceram Powerfill IVB	Nanohybrid	Ivoclar Vivadent AG, Schaan, Liechtenstein	Barium glass, ytterbium, trifluoride, copolymer, mixed oxide (79 wt%, 53–54 vol%)	Bis-GMA, Bis-EMA, UDMA, Bis-PMA, DCP, D3MA.	Camphorquinone, tertiary amines, Ivocerin, TPO

Bis-GMA—bisphenol A-diglycidyl dimethacrylate; Bis-EMA—ethoxylated bisphenol A dimethacrylate; UDMA—urethane dimethacrylate; TEGDMA—triethylene glycol dimethacrylate; PEGDMA—polyethylene glycol dimethacrylate; Bis-PMA; Propoxylated bisphenol A-dimethacrylate; DCP—tricyclodecane-dimethanol dimethacrylate; D3MA—decandiol dimethacrylate.

**Table 2:** Values of microhardness with standard deviation (MPa) and mean reduced modulus with standard deviation (MPa) of commercial dental composites; samples were cured on one side—top of the sample. Distance is the depth of layer presented in µm.

Distance (µm)	Filtek Ultimate				Estelite Σ Quick				Tetric EvoCeram BLXL				Tetric EvoCeram Powerfill IVB			
	Microhardness (MPa)		Reduced Modulus (MPa)		Microhardness (MPa)		Reduced Modulus (MPa)		Microhardness (MPa)		Reduced Modulus (MPa)		Microhardness (MPa)		Reduced Modulus (MPa)	
0	300	±90	8760	±580	420	±30	6630	±560	590	±0	11,510	±190	650	±140	10,560	±1450
675	2440	±530	25,180	±3350	790	±20	11,100	±290	1180	±160	20,370	±2180	1190	±70	20,120	±500
1250	1570	±160	17,770	±1000	860	±200	12,020	±720	1260	±200	21,410	±3150	1250	±170	20,810	±4450
1925	900	±120	9400	±890	110	±40	1430	±260	880	±90	15,540	±1130	1130	±180	19,920	±1220
2600	400	±30	5910	±240	80	±0	1690	±210	140	±70	4010	±1030	270	±80	3790	±780

**Table 3:** The mean radial (σr), circumferential (σθ) and contraction stress generated during photopolymerization (σint) expressed in MPa with standard deviation.

Dental Composite	σr		σϴ		σint	
	(MPa)		(MPa)		(MPa)	
Filtek Ultimate	6.1	±0.4	−7.6	±0.5	13.7	±0.8
Estelite Σ Quick	4.3	±0.4	−5.7	±0.5	10	±0.9
Tetric EvoCeram BLXL	3.9	±0	−5.3	±0.1	9.1	±0.1
Tetric EvoCeram PowerFill	3	±0.5	−4.4	±0.5	7.4	±1

## Data Availability

The data presented in this study are available on request from the corresponding author.

## Appendix A

**Table A1:** Median values of Vicker’s hardness, diametral tensile strength (DTS), three-point bending flexural strength (TPB) and modulus of elasticity in bending (FS modulus) with standard deviation for tested commercial composites.

Composite	Vicker’s Hardness (HV)	DTS (MPa)	FS (MPa)	FS Modulus (MPa)
Filtek Ultimate	93.82 ± 17.44	48.03 ± 5.97	87.32 ± 19.03	6720 ± 791.5
Estelite Σ Quick	52 ± 3.92	31.05 ± 4.25	64.6 ± 6.65	4491 ± 514.8
Tetric EvoCeram Bleach	58.82 ± 7.33	32.9 ± 3.53	63.58 ± 7.53	5450 ± 318.04
Tetric EvoCeram Powerfill	54.27 ± 4.1	32.5 ± 5.29	79.3 ± 14.37	7514 ± 1276.61
